# Brain-derived neurotrophic factor promotes bone regeneration in a canine model of peri-implantitis

**DOI:** 10.1186/s40729-024-00580-9

**Published:** 2024-11-26

**Authors:** Shoko Kono, Shinya Sasaki, Shinji Matsuda, Katsuhiro Takeda, Tomoyuki Iwata, Kazuhisa Ouhara, Mikihito Kajiya, Hidemi Kurihara, Noriyoshi Mizuno

**Affiliations:** 1 Department of Periodontal Medicine, Graduate School of Biomedical and Health Sciences, Hiroshima University, 1-2-3, Kasumi, Minami-Ku, Hiroshima, 734-8553 Japan; 2 Department of Biological Endodontics, Graduate School of Biomedical and Health Sciences, Hiroshima University, Hiroshima, Japan; 3 Center of Oral Clinical Examination, Hiroshima University Hospital, Hiroshima, Japan

**Keywords:** BDNF, Peri-implantitis, Bone regeneration, Reosseointegration, Canine model

## Abstract

**Purpose:**

The present study aims to determine whether the brain-derived neurotrophic factor (BDNF)/high-molecular-weight hyaluronic acid (HMW-HA) complex could regenerate bone around implants lost due to peri-implantitis.

**Methods:**

Dogs had their three premolars extracted, and three implants were placed on each side. After osseointegration, 3-0 silk threads were ligated around the healing abutment for 12 weeks. Implants were classified into four groups—no treatment (control group), non-surgical debridement (debridement group), non-surgical debridement with application of HMW-HA (HMW-HA group), and non-surgical debridement with application of BDNF/HMW-HA complex (BDNF/HMW-HA group). Probing pocket depth (PPD), attachment level (AL), and bleeding on probing (BOP) were recorded before and 12 weeks after each treatment. Standardized intraoral radiographs were obtained, and histological analysis was conducted.

**Results:**

The bone level on radiographs significantly improved (median −0.15 mm, IQR −0.31 to 0.10) only in the BDNF/HMW-HA group, while changes in PPD and AL were similar to those in other groups. The BOP positivity rate decreased in the debridement and BDNF/HMW-HA groups. Unlike images of the other groups, histological images of the BDNF/HMW-HA group showed no epithelial migration toward the tip of the implant. Inflammatory cell infiltration was reduced compared with that in the other groups. New bone was observed around the implants only in the BDNF/HMW-HA group.

**Conclusions:**

The BDNF/HMW-HA complex appears to promote bone regeneration when combined with non-surgical debridement for peri-implantitis.

## Background

Peri-implantitis is an inflammatory condition of the tissue surrounding dental implants, with progressive resorption of the supporting bone [1]. The current treatment for peri-implantitis recommends an initial non-surgical approach, followed by surgical decontamination when this approach is not sufficiently successful [2]. In addition to flap surgery, which primarily focuses on decontaminating the infectious implant surface, many regenerative approaches have been attempted. However, no procedures or materials for bone augmentation have been confirmed to improve long-term prognosis [3]. The guidelines also state that no evidence demonstrates the superiority of any specific surgical technique [2]. Therefore, establishing a bone regenerative method that improves long-term prognosis is necessary.

Brain-derived neurotrophic factor (BDNF) is a growth factor involved in the survival and differentiation of neural cells [4]. It primarily binds to two receptors—tropomyosin receptor kinase B (TrkB), a high-affinity receptor, and p75, a low-affinity receptor [5]. These receptors are expressed on various cells other than neurons, including osteoblasts [6]. BDNF has been shown to enhance fracture healing by promoting osteoblast migration and integrin β1 expression. This is carried out by activation of the ERK1/2 and AKT signaling pathways via TrkB [7]. BDNF has also been shown to promote osteoblast differentiation of mesenchymal stem cells (MSCs) cultured on titanium alloy in vitro [8]. To establish osseointegration, bone marrow-derived MSCs need to migrate to the implant body surface and differentiate into osteoblasts [9]. We speculated that a similar process may be needed to re-establish osseointegration disrupted by peri-implantitis and that BDNF may influence MSCs and osteoblasts to promote re-osseointegration.

We previously reported that BDNF induces periodontal tissue regeneration without surgical procedures in a canine experimental periodontitis model [10]. We performed scaling and root planing and injected a mixed gel of BDNF and high-molecular-weight hyaluronic acid (HMW-HA) into the periodontal pocket. Because HMW-HA has anti-inflammatory effects and its viscosity can be changed freely, we used it as a carrier for BDNF. The capacity of HMW-HA to sustainably release BDNF has also been verified in vitro [11]. We hypothesized that bone regeneration around implants could be achieved using a BDNF/HMW-HA complex in a similar manner through the effects of BDNF on osteoblasts and MSCs.

## Methods

### Experimental animals

Ten female beagles (aged 12–20 months, 10–14 kg) were used (Table 1). Healthy dogs without missing teeth were included in this study. The dogs were housed in individual cages at 22 ± 2 °C with a 12-h light/dark cycle (lights on at 8 a.m., lights off at 8 p.m.). Food and water were provided ad libitum. All animal experimental procedures were approved by the Committee of Research Facilities for Laboratory Animal Science at the Hiroshima University School of Medicine (approval no. A16-140). Additionally, all animal experiments complied with the ARRIVE guidelines.

**Table 1:** Number of experimental units

Group	n (measurement points)	Included implants	Excluded implants	Animals
Control	20	5	1	2
Debridement	44	11	1	4
HMW-HA	28	7	5	3
BDNF/HMW-HA	60	15	3	5
Peri-implantitis model^a^	24	6	0	1
Intact implant^b^	–	4	2	1
Total	176	48	12	10

**Fig. 1:**
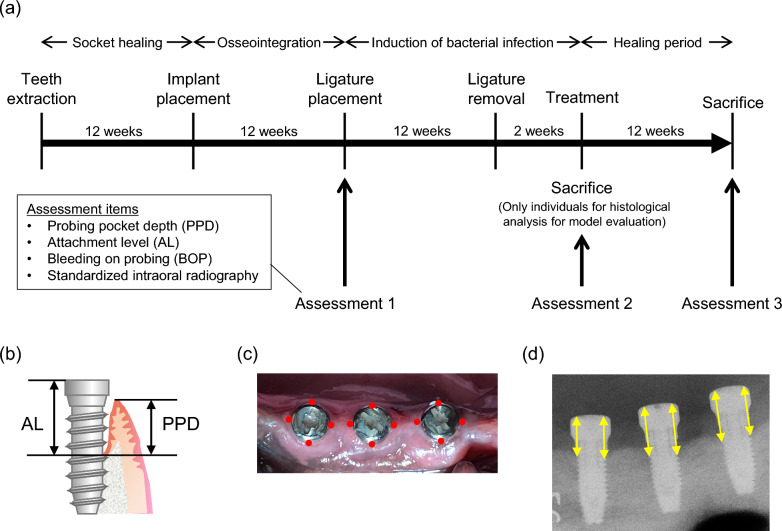
Experimental design and methods of assessment. **a** Outline of the experiment. Oral hygiene was performed two times a week during the osseointegration and healing periods. To promote biofilm formation, a soft diet was provided during the ligation period. **b** Definition of probing pocket depth (PPD) and attachment level (AL). PPD is the distance from the margin of peri-implant mucosa to the bottom of the pocket, measured with a probe. AL is the distance between the top of the healing abutment and the bottom of the pocket, measured with a probe. **c** Measured position on probing. For each implant, measurements were taken by inserting a probe at the following four points: mesial, distal, buccal, and lingual. **d** Measurement method of intraoral radiography. On the radiography measurement using ImageJ, the length between the top of the healing abutment and the top of the bone on both mesial and distal sides for each implant was measured. PPD, periodontal probing depth; AL, attachment loss; BOP, bleeding on probing

The number of implants used in the experiment is displayed in the "Included implants" column. Measurements by probing were performed at four points per implant (Fig. 1c), and the number of measurement points is shown as n (measurement points)

^a^Implants used to evaluate an experimental peri-implantitis model are described

^b^Implants used to examine the appropriateness of implant placement itself, which had no ligation, are described

*BDNF* brain-derived neurotrophic factor, *HMW-HA* high-molecular-weight hyaluronic acid

### Creation of an experimental peri-implantitis model

In this study, treatment groups were randomly assigned using a random number generator in Microsoft Excel, with only one person aware of the group assignments. All surgical procedures were conducted by a single periodontist, who was blinded to the group assignments. Those responsible for daily feeding, cleaning, and oral hygiene were also unaware of the group assignments.

The dogs were sedated via intramuscular injection of a mixture of midazolam (Sandoz, Tokyo, Japan) (0.15 mg/kg), medetomidine hydrochloride (Orion, Espoo, Finland) (20 µg/kg), and butorphanol tartrate (Meiji Seika, Tokyo, Japan) (0.1 mg/kg) during all procedures and assessments. Before the procedure, all teeth were scaled to remove the source of inflammation in the oral cavity. One week after scaling, the mandibular second, third, and fourth premolars on both sides were extracted under local anesthesia using 2% lidocaine with 1:80,000 adrenaline (Dentsply Sirona, Tokyo, Japan). A period of 12 weeks was allowed for socket healing before implant placement. All teeth were scaled again, and implant surgery was conducted; after making a crestal incision, full-thickness flaps were raised, and three implants (ɸ3SETiO^®^ Plus, GC, Tokyo, Japan) were placed on each side according to the manufacturer’s instructions. The length of the implant body was 8 mm, and the total length when the healing abutment was attached was 10.5 mm. Healing abutments were mounted immediately after implant placement. The flaps were repositioned and sutured using a 5-0 nylon suture (GC). Oral hygiene was maintained by brushing two times weekly and scaling once every 4 weeks. After 12 weeks of osseointegration, the following assessments were performed (Assessment 1 in Fig. 1a): probing pocket depth (PPD), attachment level (AL), bleeding on probing (BOP), and standardized radiography. AL was defined as the length from the bottom of the pocket to the top of the healing abutment, as measured using a plastic probe (GC) (Fig. 1b). PPD and AL were measured to the nearest 1 mm. PPD, AL, and BOP were measured at four locations per implant (mesial, distal, buccal, and lingual) (Fig. 1c). To standardize the intraoral radiographs, softened paraffin wax was molded into a block (1 × 1 × 5 cm) and used to make an impression of the healing abutments. By attaching a radiographic indicator (Hanshin Technical Laboratory, Hyogo, Japan) to the wax block, the indicator could be fixed in the same position in the mouth. This ensured that the radiographic device, implants, and the X-ray film were always at the same distance and angle. According to the results of Assessment 1, implants with a PPD of ≥ 5 mm were considered to have failed and were excluded from subsequent experiments. Immediately after Assessment 1, 3-0 silk threads were wrapped around each healing abutment five times and ligated. Following this, a soft diet was provided to promote plaque adhesion around the implants. Ligatures were maintained for 12 weeks and subsequently removed. Assessment 2, similar to Assessment 1, was conducted 2 weeks after ligature removal to avoid temporary exacerbation of inflammation due to ligature removal from affecting the assessment. One dog was sacrificed without ligation to assess the adequacy of implant placement, and the other was sacrificed without being assigned to a treatment group for histological evaluation of peri-implantitis (Table 1).

### BDNF application to the peri-implantitis model

Recombinant human BDNF (R&D Systems, Minneapolis, MN, USA) and a synthesized HMW-HA gel (DENKA, Tokyo, Japan) (molecular weight: 2 million Daltons) were mixed and used. The peri-implantitis model was randomly assigned to one of four groups—control, debridement, HMW-HA, or BDNF/HMW-HA. Models were assigned such that the three adjacent implants were in the same group. The control group received no treatment. In the debridement group, debridement without flap reflection was performed using an ultrasonic scaler equipped with a polyetheretherketone tip. In the HMW-HA group, in addition to the debridement described above, HMW-HA gel was injected into the peri-implant pocket using a blunt needle. In the BDNF/HMW-HA group, BDNF/HMW-HA complex gel (BDNF: 500 µg/mL) was injected after debridement in the same manner as that in the HMW-HA group. The gel was injected into the peri-implant pocket to slightly overflow (approximately 50 µL each). After each treatment, the dogs continued to receive oral hygiene treatments (brushing and scaling) and were fed a normal diet. Assessment 3 was conducted 12 weeks after the treatment.

### Tissue preparation and histological analysis

After intraoral assessment (Assessment 3), the anesthetized dogs were fixed by perfusion with a 10% formalin solution (FUJIFILM Wako Pure Chemical Corporation, Osaka, Japan). The implants were trimmed together with the surrounding tissue and embedded in methyl methacrylate, and polished specimens were prepared with a thickness of 40 µm. Specimens were stained with hematoxylin and eosin (H&E).

### Intraoral radiographical analysis

Radiographs obtained during each assessment were analyzed using ImageJ software (National Institute of Health). The distance between the uppermost end of the bone in contact with the implant body and that of the healing abutment was measured on the mesial and distal sides (Fig. 1d). Therefore, the number of measurement points was twice the number of implants. Because the purpose of this study was bone regeneration, the change in bone level measured using this method was defined as the primary outcome.

### Statistical analysis

Data were analyzed using the Mann–Whitney U test for PPD, AL, and bone level (Table 2), the chi-square test for BOP (Tables 2 and 3), and the Steel–Dwass test for PPD, AL, and bone level (Table 3). Statistical significance was set at *P* < 0.05. All analyses were performed using JMP Pro 17. We selected a small sample size because of the exploratory nature of this study; the sample size was determined based on a previous study [12].

## Results

### Induction of peri-implantitis by ligation

**Fig. 2:**
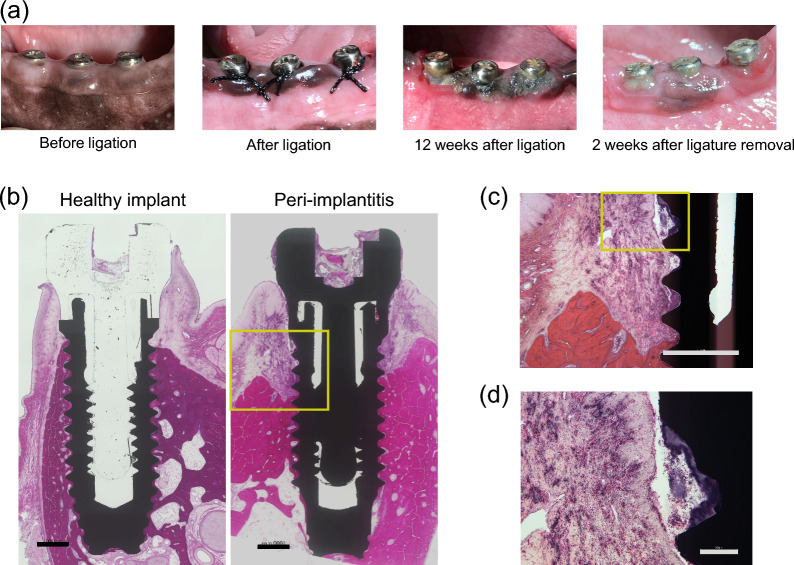
Creation of an experimental peri-implantitis model. **a** Intraoral images of the ligature-induced peri-implantitis model. **b–d** Hematoxylin & eosin staining images of non-decalcified polished specimens in healthy implant and ligature-induced peri-implantitis model. **b** Low-magnification. Scale bars = 1000 µm. **c** High-magnification image of the surrounding area in (**b**). Scale bar = 1000 µm. **d** High-magnification image of the surrounding area in (**c**). Scale bar = 200 µm

No signs of inflammation were found in the mucosa surrounding the healthy implants before the induction of peri-implantitis. However, 12 weeks after ligation, obvious mucosal swelling and redness were observed (Fig. 2a). Pocket probing revealed that PPD, AL, BOP positivity rate, and bone level all increased after ligation (Table 2). H&E-stained images of the healthy implant showed that osseointegration was established from the bottom of the implant body to the top of the screw threads, and inflammation in the peri-implant mucosa was not obvious (Fig. 2b). In contrast, osseointegration was lost and inflammatory cell infiltration was observed around the affected implant (Fig. 2b, c). Hematoxylin-stained dental calculus was also observed on the surfaces of the implant threads (Fig. 2d).

**Table 2:** Assessment of peri-implantitis model

	Before ligation	After ligation	*P*
PPD (mm) Median, IQR, Range	2 (1–3, 1–4)	3 (3–4, 1–6)	< 0.001
AL (mm) Median, IQR, Range	3 (2–4, 1–6)	5 (5–5, 3–7)	< 0.001
BOP positivity rate (%)	36.8	88.6	< 0.001
Bone level (mm) Median, IQR, Range	4.18 (3.84–4.57, 2.80–5.70)	5.51 (5.26–5.88, 4.62–6.83)	< 0.001

Before ligation corresponds to Assessment 1 in Fig. 1a, and after ligation corresponds to Assessment 2

*PPD* probing pocket depth, *AL* attachment level, *BOP* bleeding on probing, *IQR* interquartile range

### Tissue regeneration by BDNF/HMW-HA

**Fig. 3:**
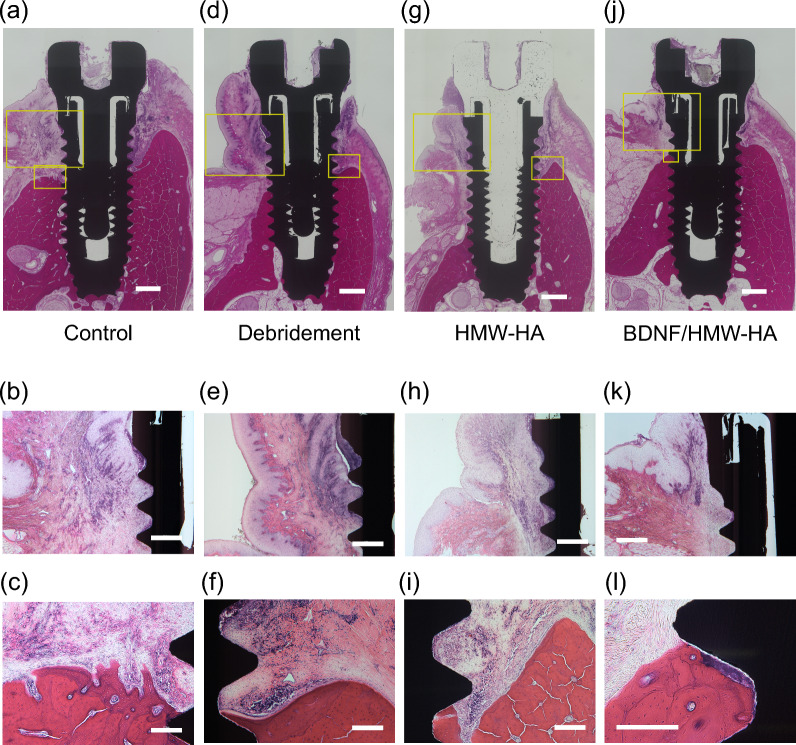
Histological examination in each treatment group. Hematoxylin & eosin staining images of non-decalcified polished specimens for each treatment group. **a**, **d**, **g**, **j** Complete images of each treatment group. Scale bars = 1000 µm. **b**, **e**, **h**, **k** High-magnification images of the larger frame in (**a**, **d**, **g**, **j**), respectively. Scale bars = 500 µm. **c**, **f**, **i**, **l** High-magnification images of the smaller frame in (**a**, **d**, **g**, **j**), respectively. Scale bars = 200 µm. HMW-HA, high-molecular-weight hyaluronic acid; BDNF, brain-derived neurotrophic factor

Only the BDNF/HMW-HA group showed improved bone levels (median −0.15 mm, IQR −0.31 to 0.10), whereas bone loss after treatment was observed in the other three groups as follows: median + 0.65 mm (IQR 0.37–0.75), median + 0.29 mm (IQR 0.10–0.63), median + 0.39 mm (IQR −0.09 to 1.47) in the control, debridement, and HMW-HA groups, respectively. Pocket probing showed that in the BDNF/HMW-HA group, both PPD and AL tended to decrease, with no significant difference from the other groups. The BOP positivity rate decreased in the debridement, HMW-HA, and BDNF/HMW-HA groups after treatment (Table 3). Histology showed severe inflammatory cell infiltration and bone resorption around the implants in the control group (Fig. 3a). In highly magnified images, the peri-implant mucosal epithelium grew toward the tip of the implant. Osseointegration was disrupted, and epithelial tissue was in contact with the implant body (Fig. 3b). In the most coronal area, where osseointegration was observed, the bone surface appeared rough and resorbed (Fig. 3c). The histological examination in the debridement and HMW-HA groups revealed loss of osseointegration and apical migration of the epithelium (Fig. 3d–i). In contrast, in the BDNF/HMW-HA group, obvious inflammatory cell infiltration was not observed in the peri-implant mucosa. Osseointegration was observed closer to the healing abutment in the BDNF/HMW-HA group than in the other groups (Fig. 3j). Epithelial migration toward the tip of the implant was not detected (Fig. 3k). Importantly, osseointegration was observed as the bone wrapped around the calculus left behind on the implant surface and extended further toward the healing abutment (Fig. 3l). This indicated that the bone in this area was newly regenerated after being lost.

**Table 3:** Assessment of each treatment group

	Control	Debridement	HMW-HA	BDNF/HMW-HA	*P*
Change in PPD (mm) Median, IQR, Range	0 (0–1, − 2 to 1)	0 (− 1 to 0, − 2 to 1)	0 (− 1 to 1, − 2 to 2)	0 (− 1 to 0, − 2 to 2)	0.073
Change in AL (mm) Median, IQR, Range	0 (0–1, − 2 to 2)	0 (0–0, − 1 to 1)	0 (0–1, − 2 to 1)	0 (− 1 to 0, − 3 to 1)	0.115
BOP positivity rate (%)					
Before treatment	80	93.2	92.9	86.7	0.369
After treatment	100	38.6	82.1	55	< 0.001
Change in bone level (mm) Median, IQR, Range	0.65 (0.37–0.75, − 0.04 to 1.24)^a^	0.29 (0.10–0.63, − 0.64 to 1.75)^b^	0.39 (−0.09 to 1.47, − 0.52 to 1.76)	−0.15 (−0.31 to 0.10, −0.85 to 0.68)^a, b^	< 0.001

Changes in PPD, AL, and bone level indicate the difference between before and after the treatment (Assessment 3–Assessment 2)

^a^ Significant difference between Control group and BDNF/HMW-HA group (*P* < 0.001, Steel–Dwass test)

^b^ Significant difference between Debridement group and BDNF/HMW-HA group (*P* = 0.004, Steel–Dwass test)

*BDNF* brain-derived neurotrophic factor, *HMW-HA* high-molecular-weight hyaluronic acid, *PPD* probing pocket depth, *AL* attachment level, *BOP* bleeding on probing, *IQR* interquartile range

## Discussion

Canine experimental models are most commonly employed to elucidate the pathology of peri-implantitis and develop treatments, because these models enable the placement of implants in the oral cavity, reproducing conditions similar to those in humans [13, 14]. Ligature models are frequently used to experimentally induce peri-implantitis [15–17]. We adopted a method previously used to induce periodontitis [10], which involved ligating silk threads around a healing abutment five times. This method is advantageous because the silk thread is less likely to fall out and requires no re-ligation. After maintaining the ligature for 12 weeks, inflammation and bone resorption around the implants were confirmed through probing, radiography, and histology, consistent with the findings in humans [1]. Furthermore, the control group’s results showed that this experimental model did not heal spontaneously; rather, inflammation persisted or progressed gradually. These findings indicate that we created a suitable model that closely mimics human peri-implantitis.

The radiographic bone level partially recovered only in the BDNF/HMW-HA group. Furthermore, a comparison of histological images showed that peri-implant mucosal inflammation was resolved in the BDNF/HMW-HA group, and the bone in contact with the implant body was located closer to the crown. These results demonstrated that the BDNF/HMW-HA complex contributes to the elimination of inflammation and bone regeneration in peri-implantitis.

Appropriate control of the inflammatory response during the initial phase of tissue regeneration is important [18, 19]. HMW-HA has anti-inflammatory effects and can be beneficial for bone regeneration [11]. According to recent reports, new bone formation can be further enhanced by HA when used as a carrier for osteoinductive growth factors, drugs, and cells [20]. HA promoted osseointegration of implants by binding to the implant surface and sustainably releasing bioactive components. However, no clear evidence exists that HA alone promotes bone regeneration [21]. Although it has been reported to affect osteoblast activity, [22] whether HA alone has the capacity for bone formation or regeneration is debatable. Considering these reports, HMW-HA alone can be concluded to have a limited effect on bone regeneration in the BDNF/HMW-HA group. Bone regeneration appears to be caused by BDNF rather than the anti-inflammatory effect of HMW-HA.

Various attempts have been made to regenerate bone defects in patients with peri-implantitis; however, no evidence exists that these methods improve the long-term prognosis [2]. Osseointegration requires bone marrow-derived MSCs to line up on the implant body surface and differentiate into osteoblasts [9]. Even during reacquiring damaged osseointegration, the remaining bone marrow-derived MSCs have been postulated to primarily contribute to bone regeneration. In this study, bone regeneration was observed only in the BDNF/HMW-HA group. BDNF has been speculated to contribute to bone regeneration around implants by promoting the proliferation and differentiation of MSCs.

In this study, we adopted a method to decontaminate the implant surface without reflecting the flap. This method is less dependent on the dentist’s skill and less invasive than surgery, which is its major advantage. However, this study was limited by the inability of non-surgical debridement to remove the source of infection completely, which may constrain the full demonstration of the effects of BDNF. Residual calculus was visible despite debridement (Fig. 3e, l). This suggests that treatment with non-surgical debridement alone is preferable only for early peri-implantitis with shallow bone defects, which are easily accessible using instruments. For deep bone defects that are difficult to reach using instruments, surgical decontamination would further enhance bone regeneration using BDNF.

Another limitation of this study is the exclusion of some implants during model creation. Although all dogs had implants placed in a standardized manner, certain implants exhibited bone resorption before ligation, likely due to occlusal trauma. Notably, the healing abutments of the excluded implants showed signs of wear, indicating occlusal trauma from the dogs biting the cage at the implant site. It is necessary to consider how to create a model in which fewer implants will be excluded, with one possibility being the use of two-stage implants to prevent occlusal trauma immediately after placement.

## Conclusions

Our results suggest that the BDNF/HMW-HA complex may promote bone regeneration when used along with non-surgical debridement to treat peri-implantitis. This study functions as a pilot study to explore new treatment methods for peri-implantitis. Further investigation is required into the mechanism of bone regeneration. Additional studies on toxicity and safety and experiments to determine appropriate concentrations are also needed. As mentioned, the method used in this study occasionally resulted in insufficient debridement of the implant surface, necessitating strict indication criteria. As a step toward clinical application, the information obtained from this study should be used as the basis for subsequent research endeavors.

## Data Availability

The datasets used and/or analyzed during the current study are available from the corresponding author on reasonable request.

## Abbreviations

AL: Attachment level
BDNF: Brain-derived neurotrophic factor
BOP: Bleeding on probing
H&E: Hematoxylin and eosin
HMW-HA: High-molecular-weight hyaluronic acid
MSCs: Mesenchymal stem cells
PPD: Probing pocket depth
TrkB: Tropomyosin receptor kinase B
